# System of Diseases of the Eye

**Published:** 1898-04

**Authors:** 


### Book Reviews.

SYSTEM OF DISEASES OF THE EYE. By American, British, Dutch, French, German and Spanish authors. Edited by WILLIAM F. NORRIS, A. M., M. D., and CHARLES A. OLIVER, A. M., M. D., of Philadelphia, Pa., U. S. A. Volume II. Examination of the eye, school hygiene, statistics of blindness and antisepsis. With thirteen full-page plates and 214 text illustrations. Octavo, pp. 556. Philadelphia: J. B. Lippincott Co. 1897.

This second volume of the System of diseases of the eye, like the first, is rich with facts and observations pertaining to the subjects of which it treats. These comprehend examination of the eye, school hygiene, blindness and its prevention and antisepsis. There are fourteen sections and each is contributed by someone whose studies and experience especially fit him to give the best and latest information on the subject which he has in hand.

The first section is On the methods for determining the acuity of vision, and is written by the learned Herman Snellen, M. D., of Utrecht, author of the almost universally used test-types, and professor of ophthalmology in the University of Utrecht, in Holland ; translated by George A. Berry, M. B., F. R. S. C., Ed., lecturer on ophthalmology in the University of Edinburgh, Scotland. It contains a concise statement, in twenty pages, of the principles upon which visual tests should be based and the various objects and methods that have been suggested for use.

Following this, his son, Herman Snellen, Jr., M. D., ophthalmic surgeon to the Netherlands hospital, Utrecht, occupies sixteen pages of the book in describing the substances, their properties and actions, known as mydriatics and myotics. The comparative value of those belonging to each class is discussed and many valuable suggestions are indicated.

The next article, also sixteen pages in length, is on Lateral illumination ; magnifying instruments employed in combination with lateral illumination ; the use of highly magnifying glasses with the ophthalmoscope, by L. Laquer, M. D., professor of ophthalmology, etc., in the University of Strasburg, Germany ; translated by Harry Friedenwald, A. B., M. D., associate professor of ophthalmology and otology, College of Physicians and Surgeons, of Baltimore, Md. This is an excellent and detailed consideration of a valuable means of diagnosis, giving its history, principles and application and the various forms of instruments that have been and are used, both separately and combined with the ophthalmoscope and magnifying glasses.

In the next twenty-six pages, George M. Gould, A. M., M. D., of Philadelphia, describes The ophthalmoscope and the art of ophthalmoscopy in a well-written and comprehensive article. It reviews the history of the ophthalmoscope, the various forms of the instrument, the methods of using it and what may be seen with it in a normal eye and in one with congenital defects.

Skiascopy (the shadow-test, retinoscopy,) and its practical application is treated by Edward Jackson, A. M., M. D., professor of diseases of the eye in the Philadelphia Polyclinic and surgeon to Wills eye hospital, Philadelphia. Twenty-two pages are given to this subject and it is clearly described by a master of the method. He embodies in the article the optical principles on which it is based, the practical application and use of the test, the apparatus needed and a brief historical sketch.

Adolphe Javal, Jr., of Paris, in thirty pages considers Ophthalmometry and its clinical application. This chapter is limited principally to the ophthalmometry, and especially to the Javal and Schiötz form and application, with an incidental reference to keratoscopy by means of a "keratoscopic disk" and to Tscherning's "ophthalmophakometer." It is an instructive article, but it seems unfortunate that "ophthalmometry" ever should have been made to signify measurements of the cornea, instead of all parts of the eye, according to the etymological sense of the word. Keratometry would be a much better term.

Prisms and prismometry, by William S. Dennett, M. D., of New York, surgeon to the New York eye and ear infirmary, occupies twenty-six pages, and is an exposition of the subject which will commend itself, both to the mathematical and non-mathematical mind. The optical principles involved are elucidated by mathematical formulæ, and the practical application of prisms in ophthalmology is clearly set forth.

Next follows a chapter of twenty-two pages by George T. Stevens, M. D., Ph. D., of New York, on The principles of and the methods for the estimation of the balance of the extraocular muscles. In this is described the various anomalies of muscular balance, together with the nomenclature which the author has devised and which is already quite generally accepted and the various methods of determining and measuring them.

The succeeding article on Perimetry and its clinical value is the most extended in the book, covering 126 pages, and is entirely out of proportion to the other articles as compared to the importance of the subjects. It is written by Herman Wilbrand, M. D., ophthalmic surgeon to the general hospital of Hamburg, Germany, and translated by Thomas R. Pooley, M. D., formerly professor of ophthalmology in the New York Polyclinic and surgeon to the New Amsterdam eye and ear hospital, New York. It is divided into a general part and a special part. In the former is described the field of vision under varying conditions and the methods used for measuring and recording it; in the latter, the significance of the special changes which take place in the visual fields in (*a*) diseases of the retina and choroid, (*b*) of the optic nerves within the orbit, (*c*) of the optic chiasm, (*d*) of the optic tracts, (*e*) of the primary optic centers, (*f*) of the intracerebral tracts leading to the cortical visual centers, (*g*) of the cortical visual centers. All these subjects, as bearing upon the field of vision, are treated in detail. The article is, indeed, a complete treatise in itself, and its study will greatly profit the general practitioner as well as the ophthalmologist.

Detection of color-blindness is a valuable article of thirty-eight pages and is written by William Thomson, M. D., professor of ophthalmology in the Jefferson Medical College and surgeon to the Wills eye hospital, Philadelphia, assisted by Carl Weiland, M. D., of Philadelphia. It is a practical article on a most important subject. Dr. Thomson has done much to bring about the practice of examining railroad employees for color-blindness, and the results of his extended experience and study are embodied in this article.

Perhaps one of the most useful and practical articles of the book is the next one on School hygiene, by S. D. Risley, A. M., M. D., Ph. D., professor of ophthalmology in the Philadelphia Polyclinic and College for Graduates in Medicine, and surgeon to the Wills eye hospital, of Philadelphia. It occupies sixty-six pages and deals with defects of vision in school children and their causes and detection, the necessity for examination of the eyes on entrance to school, the need of correction of refractive errors in school children, and the hygiene of the school-room, including lighting, seating and posturing. The whole article is full of wise suggestions and the following of them would do much to prevent eye diseases and the manifold disturbances arising from eye-strain.

Another important article is the next one, of forty-four pages, on Blindness: its frequency, causes and prevention, by I. Minis Hays, A. M., M. D., of Philadelphia. It first deals with statistics and the relation of climate, age and sex to blindness. Then follow sections on congenital blindness, blindness due to idiopathic diseases of the eye, blindness due to traumatism, blindness due to general diseases, the influence of occupation on eye disease and the prevention and diminution of blindness. Such a survey by such a writer can but impress the reader that preventive measures are of prime importance in diseases of the eye, as well as in diseases of other parts of the body.

The two concluding articles are on Antisepsis, twenty-six pages, by Joseph A. Andrews, M. D., ophthalmic surgeon to the Charity hospital, New York, and The microörganisms of the conjunctiva and lachrymal sac, fifty-two pages, by Joseph McFarland, M. D., demonstrator of pathological histology in the University of Pennsylvania, and Samuel Stryker Kneass, M. D., adjunct professor of bacteriology in the Philadelphia Polyclinic and School for Graduates in Medicine. The articles present an excellent résumé of the bacteriology of the conjunctiva and lachrymal sac and of the methods and limitations of antisepsis as applied to the eye.

The volume, as a whole, maintains a high standard of excellence, both as regards the matter, the illustrations and the general make-up. Like its predecessor, it can but take high rank in ophthalmological literature. A. A. H.

